# Hypernatremia in Diabetic Ketoacidosis: A Rare Metabolic Derangement Requiring a Cautionary Approach in Fluid Resuscitation

**DOI:** 10.7759/cureus.36689

**Published:** 2023-03-26

**Authors:** Pooya Zardoost, Zeryab Khan, Henry L Wehrum, Ryan Martin

**Affiliations:** 1 Graduate Medical Education, OhioHealth Doctors Hospital, Columbus, USA

**Keywords:** covid and dka, dka, lactated ringers, covid-19, community aquired pneumonia, pneumonia, anion-gap metabolic acidosis, fluid treatment, hypernatremia, diabetic keto acidosis

## Abstract

Diabetic ketoacidosis (DKA) with hypernatremia is an atypical metabolic derangement that warrants additional consideration in choosing IV fluids. Our patient, a middle-aged male with a history of insulin-dependent diabetes mellitus type 2 and hypertension, presented with DKA and hypernatremia in the setting of poor intake, community-acquired pneumonia (CAP), and COVID-19. DKA and hypernatremia led to a meticulous approach to fluid resuscitation, where a crystalloid solution was the choice in treating and preventing exacerbation of either condition. Successful treatment of these conditions requires understanding the unique pathophysiology, which demands further research on management.

## Introduction

Typically, in diabetic ketoacidosis (DKA), factitious hyponatremia presents as an electrolyte disturbance [1]. Even after correcting the glucose elevation, there is a risk of overcorrecting the hyponatremic state to osmotic demyelination syndrome [2]. However, our patient had DKA with hypernatremia, which required a tactful fluid resuscitative approach to manage both metabolic derangements successfully. This article was previously presented as a meeting abstract at the 2022 ACP Ohio and US Air Force Chapters Scientific Meeting on October 21, 2022.

## Case presentation

A man in his early 50s with a past medical history significant for type-2 diabetes, hypertension, and stroke presented to the ED with altered mental status. The patient was unresponsive, and history was obtained primarily from medics who responded to a call from the patient’s family. The family stated that the patient had been experiencing generalized malaise and cold-like symptoms for two weeks, and his symptoms worsened several days prior to admission to the point that he could not get out of bed. They also reported that he had not been following his regular insulin regimen since then. It was unclear if he had been taking the rest of his home medications of atorvastatin, hydrochlorothiazide, and metformin.

In the ED, the examination was significant for altered mental status with a Glasgow Coma Score of 10. Vital signs and physical exam reflected tachycardia, tachypnea akin to a Kussmaul respiratory pattern, mild hypotension, and oxygen desaturation.

The patient’s vitals and labs met the criteria for sepsis, and he tested positive for COVID-19. Urine antigen was positive for *Streptococcus pneumoniae*. His metabolic profile was significant for blood glucose of 1,580 mg/dL, a corrected sodium level of 172 mmol/L with 17 L free water deficit, an anion gap > 40 mmol/L, serum creatinine of 4.68 mg/dL, potassium of 6.5 mmol/L, and lactic acid of 7.6 mmol/L. Given this data, a venous blood gas and beta-hydroxybutyrate (BHB) level were collected and reported a pH of 7.19 and BHB of 10.1 mmol/L, indicating severe DKA.

The patient was placed in COVID-19 isolation. He received three liters of a bolus of normal saline, and after blood cultures were drawn, he was initiated on broad-spectrum antibiotics with cefepime and vancomycin. Chest X-rays were notable for bilateral lower lobe infiltrates, and after urine, *Streptococcus pneumoniae* returned positive. Antibiotics were de-escalated to doxycycline and azithromycin. The patient’s oxygenation initially improved on bilevel positive airway pressure (BiPAP), but due to severe metabolic derangement and acute encephalopathy, intubation was required, and the patient was admitted to the ICU. He was diagnosed with DKA, severe sepsis, COVID-19, and hypernatremia. Enteral nutrition was initiated with nutritional supplements at 75 ml/hr, which provided 1650 kcals that consisted of 152 grams of protein, 125 grams of carbohydrates, and 1386 ml of free water. Nephrology was consulted, and the patient was given two additional liters of electrolyte-A IV solution and then started on half normal saline at 125 ml/hr as well as IV insulin infusion with free water flushes via nasogastric tube (NGT) with a sodium decrease goal of 6 mmol/L each day, and total fluid goal of 2600 ml daily. The patient’s sodium slowly improved after the first 24 hours of admission, and by the third day, the patient’s sodium level dropped roughly by 10 milliequivalents from the initial presentation. After a decrease in glucose below 250 mg/dL, the patient was transitioned to dextrose 5% in water (D5W) per DKA protocol.

After nine days, the patient’s corrected sodium improved to 143 mmol/L, and his mentation improved. The D5W was stopped, and the patient was successfully liberated from the ventilator, nasogastric tube, and foley catheter with transfer from the ICU to continued care on the step-down floor. He was noted to be volume overloaded, which was resolved with administrations of furosemide and chlorothiazide. For the remainder of his hospitalization, the patient was transitioned to a basal-bolus insulin regimen and removed from COVID-19 isolation. Due to deconditioning from his prolonged hospitalization requiring mechanical ventilation, the patient was discharged to a skilled nursing facility for two weeks. His home insulin regimen was modified to 50 units of insulin glargine twice daily, and he was to continue his 15 units of insulin aspart with each meal. The rest of his home medications were continued and unchanged.

## Discussion

Our patient presented with several life-threatening metabolic disturbances, including hyperglycemia, acidosis, and hypernatremia in the setting of COVID-19 with concomitant CAP. His infection with COVID-19 and CAP, combined with not adhering to his insulin regimen, served as the inciting factor to his hyperglycemic crisis and diagnosis of DKA. Data supporting this diagnosis included his severely elevated glucose on admission and further labs displaying ketosis and acidosis. The diagnosis of hypernatremia was revealed through high corrected sodium. DKA is diagnosed via a combination of metabolic acidosis, increased total body ketones, and hyperglycemia [3,4]. While most patients diagnosed with DKA have type 1 diabetes, it can also be seen in those with type 2 diabetes [5]. Our patient presented with dehydration, encephalopathy, and Kussmaul breathing, which are clinical hallmarks of DKA [3,4]. With a pH less than 7.3, a blood glucose greater than 250 mg/dL, an anion gap of more than 12 mmol/L, bicarb under 18 mmol/L, and elevated BHB, his metabolic profile met the criteria for DKA [3,4]. The etiology of DKA can be connected to several precipitants, including infection, trauma, and medication non-compliance [3,4]. Our patient’s CAP with concomitant COVID-19 infection and insulin non-compliance acted as the precipitating factors leading to a progressively insulin-deficient state [3,4]. Increases in counter-regulatory hormones such as cortisol, catecholamines, and glucagon lead to the production of keto-acids [3,4]. In addition to DKA and infection, the patient had an additional life-threatening metabolic abnormality in the form of hypernatremia. After correcting for glucose, his sodium was elevated at 172 mmol/L. Sodium is a crucial osmotic solute that plays the primary role in intracellular and extracellular homeostasis [2]. Treatment of hypernatremia consists of D5W with the goal of lowering serum sodium, but not lowering by more than 12 mmol/L in the first 24 hours [6]. These patients are at risk of cerebral edema if the sodium is corrected too rapidly [6].

In DKA, patients typically present with a metabolic profile that points to hyponatremia, but this is viewed as factitious hyponatremia as the elevated blood glucose acts as the predominant osmole in these patients, which lowers the relative concentration of sodium despite no changes in actual sodium levels [7]. Clinical practice based on conventional literature recommends correcting the true sodium level by approximately 1.6 mmol per liter for every 100 mg/dL above 100 mg/dL of glucose [7]. Our patient presented with hypernatremia, which further complicated management.

The etiology of our patient’s hypernatremia is multifactorial, though several of these factors require further research. While poor intake and deconditioning from an infection likely played a role, it is still uncommon for a DKA patient to present with hypernatremia. The proposed mechanism for hypernatremia in DKA emanates from excessive osmotic diuresis that leads to a volume-depleted state [8]. The patient’s home regimen included hydrochlorothiazide. While it is unclear if the patient was compliant with this medication, there may have been additional volume depletion if he had taken it. A retrospective study in 1997 examined DKA cases at a pediatric hospital from 1992 to 1994 and concluded that hypernatremia with DKA led to prolonged ICU stays [9]. Patients with new-onset diabetes were more likely to have hypernatremia than those who were previously known to be diabetic [9]. It has been proposed that prolonged DKA eventually leads to loss of hypotonic urine with increased glomerular filtration rate [8,9]. In pediatric populations, the presence of hypernatremia in DKA has been associated with the ingestion of carbohydrate-rich soft drinks [10]. In our case, it was unclear how long our patient had been non-compliant with his insulin regimen or his diet. However, based on the patient’s altered mentation, paired with his hyperglycemia on presentation, it is possible that this patient was initially hyponatremic but gradually transitioned to hypernatremia after the loss of hypotonic urine. Also, the patient was acidotic, which has been correlated with the level of altered mentation in DKA [11]. It may be possible that our patient’s altered mentation also contributed to less ingestion of water, underscored by the total water deficit of 17 liters. Apart from the 1997 study mentioned above, current data on hypernatremia in the setting of DKA, especially in the adult population, are limited to case series and case reports, in which fluid regimens varied greatly from Ringer’s Lactate, half-normal saline, and even normal saline [8, 11, 12].

The second component further adding to the need for research is the patient’s precipitating infections with COVID-19 and *Streptococcus pneumoniae*. His accompanying respiratory symptoms diagnosed as COVID-19 and CAP may have also played a role in not just the initial insult that led to DKA but also the concomitant hypernatremia prolonging and complicating his stay. COVID-19 has been associated with a wide spectrum of electrolyte abnormalities. A 2021 study of 9,946 patients hospitalized for COVID-19 found that hyponatremia was more common among COVID-19 patients than hypernatremia. Hypernatremia was strongly associated with in-hospital deaths [13,14]. As research into the manifestations of COVID-19 remains dynamic, additional emphasis must be placed on electrolyte abnormalities. While our patient was fully vaccinated for COVID-19 the previous year, the bacterial co-infection may have played a role in amplifying the severity of his signs and symptoms. A meta-analysis in 2021 reported that approximately 7% of patients hospitalized with COVID-19 had a bacterial co-infection, and a higher proportion of COVID-19 patients in the ICU had bacterial co-infections [15]. In previous pandemics and seasonal influenza, bacterial co-infection is associated with more severe outcomes [15].

Lastly, no consensus exists regarding fluid resuscitation for hypernatremia in the DKA setting. Initially, we experienced challenges in fluid resuscitation, with three liters of normal saline already being administered. D5W is a hypotonic solution given to help correct hypernatremia, but the patient’s uncontrolled glucose did not make this a reasonable option [6]. Half-normal saline has been tried in previous cases, and the patient was eventually transitioned to this solution for maintenance. However, caution must be exercised in decreasing the serum osmolality too fast with the low sodium content of this fluid choice, which, accounting for insensible losses and the 17 L free water deficit in our patient, may have caused cerebral edema [8]. The initial boluses of electrolyte-A IV solution may have played a driving role in the steady decrease in our patient’s sodium. Electrolyte-A IV solution is an isotonic buffered IV solution with a physicochemical profile similar to human plasma [16,17]. Data is evolving on the potential benefits of balanced crystalloid solutions such as Ringer’s Lactate and electrolyte-A IV solutions in patients with DKA. A 2020 subgroup analysis of cluster randomized control trials found that balanced crystalloids led to a more rapid DKA resolution than saline [18]. These balanced crystalloids have a chloride concentration similar to that of the body, which may influence clinical decision-making in avoiding hyperchloremic metabolic acidosis in DKA patients who, by clinical criteria, are already in high-anion gap acidosis. However, in some of the cases of hypernatremia and DKA, non-balanced fluids such as normal saline continued to be used, and even among the balanced fluids, some differences may have a clinical impact in treating both metabolic derangements. Electrolyte-A IV Solution has a sodium content of 140 milliequivalents, which is 10 milliequivalents more than the sodium content of Ringer’s Lactate; whether this difference in sodium content has significance in the degree of correcting hypernatremia in the setting of DKA is unclear [19].

## Conclusions

Frequent surveillance of metabolic status, particularly the sodium and anion gap, influenced the choice of fluid therapy, how aggressively we could provide boluses, and how the rate of IV maintenance fluids could be modified. Fortunately, through these adjustments, the patient recovered. In our practice, this case lends to considering a balanced crystalloid fluid approach to patients with an etiology of DKA triggered by a precipitating factor and combined with hypernatremia precipitated by poor intake. The fluid treatment regimens for DKA and hypernatremia generally consist of normal and/or half-normal saline and D5W, respectively. However, when both conditions are present, additional consideration should be given to crystalloid solutions closer to the physicochemistry of human plasma. More data is necessary on fluid management with hypernatremia and DKA, as well as how comorbidities and compliance history can impact the length of hospital stay in the adult population. While factitious hyponatremia is more common in DKA due to the hyperosmolar state of DKA, clinicians should be mindful of this less common presentation and utilize interdisciplinary specialties when managing fluid resuscitation.
